# The public health implications of antiretroviral therapy – 2011 and beyond

**DOI:** 10.1186/1758-2652-13-S4-K2

**Published:** 2010-11-08

**Authors:** KM De Cock

**Affiliations:** 1 Center for Global Health, Centers for Disease Control and Prevention, Atlanta, GA, USA

## 

2011 will mark the 30^th^ anniversary of the first description of AIDS, and the 15^th^ year since combination antiretroviral therapy (ART) was introduced. Since its introduction, ART has had profound impact on AIDS incidence and mortality in industrialized countries, and international collaboration, mainly through the Global Fund and the President’s Emergency Plan for AIDS Relief, has provided treatment to more than 4 million HIV-infected persons in low and middle income settings.

Despite these advances and much speculation, the full prevention benefits of ART still await clarification. Experience with mother-to-child transmission of HIV gave insight into the critical importance of HIV RNA as the dominant risk factor for HIV transmission, as well as proof of concept that ART reduced transmission rates through lowering viral load. Important studies published in 2010 include evidence that among couples discordantly infected with HIV, a 92% reduction in transmission occurred when the infected index was taking ART (Donnell D, et al. Lancet 2010). The concept has arisen of community viral load as a risk factor for community-wide transmission (Das-Douglas, CROI 2010), and ecologic evidence has been presented of ART scale-up correlating with reduced HIV incidence at the population level (Lima VD, Lancet 2010). These observations suggest more widespread ART among HIV-infected persons (“test and treat”) could provide substantial prevention benefit but data on such an approach are awaited, despite several mathematical models examining this.

ART may provide prevention benefit when given as post-exposure prophylaxis and possibly as pre-exposure prophylaxis. The recent widely acclaimed study of a tenofovir-containing gel, associated with 39% protective efficacy against HIV acquisition in women on primary analysis (Karim QA, Science 2010), provides evidence of efficacy of pre-exposure prophylaxis as well as the feasibility of an efficacious microbicide. Results of trials of oral pre-exposure prophylaxis will become available in the near future.

These developments occur in a global context of financial economic downturn, increased attention to the other health-related millennium development goals, and an emerging pandemic of non-communicable diseases. How to use ART most effectively and comprehensively for HIV prevention will challenge decision makers, even as evidence for individual approaches mounts.

